# Caco-2 Cell Line Standardization with Pharmaceutical Requirements and In Vitro Model Suitability for Permeability Assays

**DOI:** 10.3390/pharmaceutics15112523

**Published:** 2023-10-24

**Authors:** Marta Kus, Izabela Ibragimow, Hanna Piotrowska-Kempisty

**Affiliations:** 1 Department of Toxicology, Poznan University of Medical Sciences, 30 Dojazd St., 60-631 Poznan, Poland; marta.kus@ethifarm.pl; 2 Research and Development Department of Ethifarm, Ethifarm Manufacturing Plant, 9 Stefana Zeromskiego St., 60-544 Poznan, Poland; izabela.ibragimow@ethifarm.pl; 3 Department of Basic and Preclinical Science, Institute of Veterinary Medicine, Nicolaus Copernicus University in Toruń, 7 Gagarina St., 87-100 Torun, Poland

**Keywords:** Caco-2 cell line, validation, drug permeability, pharmaceutical requirements, biowaiver

## Abstract

The Caco-2 cell line derived from human colon carcinoma is commonly used to assess the permeability of compounds in in vitro conditions. Due to the significant increase in permeability studies using the Caco-2 cell line in recent years, the need to standardize this biological model seems necessary. The pharmaceutical requirements define only the acceptance criteria for the validation of the Caco-2 cell line and do not specify the protocol for its implementation. Therefore, the aim of this study is to review the conditions for permeability studies across the Caco-2 monolayer reported in the available literature concerning validation guidelines. We summarized the main aspects affecting the validation process of the Caco-2 cell line, including the culture conditions, cytotoxicity, cell differentiation process, and monolayer transport conditions, and the main conclusions may be useful in developing individual methods for preparing the cell line for validation purposes and further permeability research.

## 1. Introduction

Oral administration of drugs is a common administration route due to its safety, ease of ingestion, and versatility to accommodate various types of drugs [1,2,3]. However, not all drugs can be administered orally due to difficulties in obtaining therapeutic concentrations, e.g., due to the first-pass effect [4,5]. Therefore, to determine the route of administration, in vitro systems are widely used to predict drug bioavailability (BA).

The BA of a drug after oral administration is largely determined by the drug’s dissolution rate, its solubility characteristics in gastrointestinal fluids, and its permeability across biological membranes (intestinal permeability) [6,7,8]. The Biopharmaceutics Classification System (BCS), as defined by Amidon et al., is a widely used tool for predicting the in vivo BA of a drug substance in in vitro conditions [9]. The BCS classifies drug substances based on their water solubility and intestinal permeability, the main factors that regulate the rate and extent of oral drug absorption [10,11]. Moreover, the BCS is widely used in the pharmaceutical industry during the processes of developing drugs and establishing their registration strategies (e.g., the biowaiver procedure in the registration process of generic drugs).

The Caco-2 (Cancer coli-2) cell line is commonly used to assess the permeability of compounds in in vitro conditions. Caco-2 cells were isolated from colon tissue derived from a 72-year-old in the 1970s [12]. Differentiated Caco-2 cells exhibit similar structural and functional properties characteristic of small intestine enterocytes [13]. Towards confluence, they begin to polarize, forming a monolayer with strong, tight junctions, apical brush borders, and microvilli. The differentiated Caco-2 cells express the typical digestive enzymes, membrane peptidases, and disaccharidases of the small intestine (lactase, aminopeptidase N, sucrase-isomaltase, and dipeptidylpeptidase IV) [14]. Therefore, the European Medicines Agency (EMA) and Food and Drug Administration (FDA) have recognized the Caco-2 cell line as a reliable in vitro model for predicting the BA of drugs in the pharmaceutical industry [10,11].

The Caco-2 cell line is characterized by high internal (the heterogeneity of cell subpopulations) [15] and external variability (e.g., resulting from intra-laboratory culture methods) [16]. Therefore, using the Caco-2 cell line in the pharmaceutical industry requires the validation of this biological model to demonstrate its suitability for the intended purposes (e.g., classification of substances into a BCS group).

According to the pharmaceutical requirements, the validation of the Caco-2 cell line is based on demonstrating an appropriate rank order relationship between the experimental permeability values of established model drugs and their absorption in human subjects [10,11]. The FDA and EMA guidelines consider the complexity and variability of biological models, thus enabling the use of individual protocols for preparing cell lines for permeability testing. However, to demonstrate the appropriate suitability and functionality of cultivated cell lines, the use of 25 model drugs is required for formal BCS studies.

The pharmaceutical requirements define only the acceptance criteria for the validation of the Caco-2 cell line and do not specify the protocol for its implementation. Therefore, the aim of this study is to review the conditions for permeability studies across the Caco-2 monolayer reported in the available literature in relation to the validation guidelines established by regulatory authorities. In this review, we presented permeability tests in the Caco-2 model used for both scientific (e.g., drug absorption prediction) and regulatory purposes (e.g., formal classification of substances into a BCS group). Moreover, we summarized the main aspects affecting the validation process of the Caco-2 cell line, including the culture conditions, cell differentiation process, and monolayer transport conditions.

## 2. General Caco-2 Validation Requirements in the Pharmaceutical Industry

According to EMA and FDA guidelines, the internal standardization of the Caco-2 cell line includes permeability studies for model compounds representing a range of in vivo human intestinal absorption, with, respectively, low (f_a_ < 50%), moderate (f_a_ = 50–84%), and high permeability (f_a_ ≥ 85%). Additionally, permeability studies of zero-permeability markers and efflux substrates should be determined [10,11]. The permeability experimental data for model drugs should be determined using the apparent permeability coefficient (P_app_) values. The P_app_ value determines the rate of transport of the substance across the Caco-2 monolayer. According to the protocols described by Tavelin [17] and Hubatsch [18], a substance is classified as a high-permeability drug if its P_app_ is higher than 10 × 10^−6^ cm/s. In turn, the low-permeability drugs are characterized by a P_app_ below 1.0 × 10^−6^ cm/s. Additionally, drugs with P_app_ values in the range of 1–10 × 10^−6^ are classified as moderate [17,18].

The validation of the Caco-2 cell line should demonstrate the correlation between the experimental transport data (P_app_) and human intestinal absorption (f_a_) of selected model drugs. To fully meet the pharmaceutical criteria, permeability assays of a minimum of five model drugs from each presented permeability group (25 model drugs in total) are required. The use of 25 model drugs during validation is intended to demonstrate the appropriate suitability and functionality of an in vitro model. Finally, a calibration curve should be developed showing the correlation between the obtained P_app_ values and the f_a_ of selected drugs with low, moderate, and high permeability [10,11]. The P_app_ values from the selected experimental Caco-2 permeability tests and the selected human absorption of all model drugs determined by the FDA and EMA are listed in Table 1.

The pharmaceutical industry guidelines specify the model drugs that may be used during the validation of the Caco-2 cell line. Eleven model drugs were established for the high-permeability group and ten markers each for the moderate- and low-permeability groups. Additionally, five and four were set for the zero-permeability and efflux substrate groups, respectively. The selection of five model drugs from each permeability group to validate the Caco-2 model is independent and can be determined individually by the researcher. Table 1 presents selected experimental data on the permeability of model drugs with high, moderate, and low permeability. The high-permeability group includes model drugs with P_app_ values in the range of 13.0–76.7 [×10^−6^ cm/s]. The low-permeability group includes substances with a P_app_ below 0.74 [×10^−6^ cm/s]. Additionally, the moderate-permeability group includes drug models with P_app_ values from 1.29 to 16.0 [×10^−6^ cm/s]. The P_app_ values for nine compounds of the moderate group are within the range of 1–10 × 10^−6^ cm/s. However, the P_app_ value (16.0 × 10^−6^ cm/s) of chlorpheniramine exceeds this range and indicates the high permeability of this substance [33]. Although chlorpheniramine is recognized by regulatory authorities as a moderate permeable drug, the permeability data of this model compound require further investigation.

## 3. Cytotoxicity Analysis of Model Compounds

The cytotoxicity of compounds on the Caco-2 monolayer is one of the crucial limitations of permeability assessments in in vitro conditions. The cytotoxic effect of the compound may lead to leaks within the Caco-2 monolayer or the reduction in transporter activities resulting, and thus to an underestimation of the efflux effect [47]. Thus, before permeability studies, it is recommended to determine the effects of all exanimated compounds on the Caco-2 monolayer’s viability.

The MTT test (3-(4,5-dimethyl-2-thiazolyl)-2,5-diphenyl-2H-tetrazolium bromide) is commonly used to determine an appropriate concentration of the analyzed substances in the Caco-2 cell line. The viability of Caco-2 cells exposed to the tested compound for a period of time equal to or longer than the conditions of the permeability test should be determined. The highest concentration of the test compounds with no cytotoxic effect on Caco-2 cells is preferred [48].

A literature review showed that the results of cytotoxicity tests of compounds on the Caco-2 cell line are not presented in detail in scientific articles. The concentrations of the compounds used in permeability tests are usually reported individually for each drug (or collectively) without detailed results of their cytotoxicity on the Caco-2 cell line. However, several available data regarding the viability of the Caco-2 cell line exposed to the BCS model drugs are presented in Table 2.

The concentrations of compounds in permeability tests through the Caco-2 monolayer should be determined by striking a balance between their solubility, cytotoxicity, and analytical response. However, in permeability studies across the Caco-2 monolayer, a wide range of concentrations from 10 [26] to 500 µM (or higher) [35,38,43] are used. The non-cytotoxic concentrations of model compounds on the Caco-2 monolayer presented in Table 2 are within this range. Therefore, the concentrations of the substances in the range of 10–500 µM seem appropriate to assess their cytotoxicity on the Caco-2 line to conduct further in vitro permeability studies. To confirm the viability of the Caco-2 monolayer after permeability tests, apoptosis assays can be additionally performed [51].

## 4. Cultivation and Maintenance of the Caco-2 Cell Line

### 4.1. Characteristics and Origin of the Caco-2 Cell Line

It is common knowledge that the Caco-2 cell line is heterogeneous and contains cells with slightly different properties [15,16]. Therefore, it is important to standardize the cultivation conditions of the cell line to retain its original properties. To minimize laboratory variability, it is recommended to use the original Caco-2 cell lines obtained from commercial banks for drug permeability testing. A summary of the commercially available Caco-2 cell lines is presented in Table 3.

Caco-2 cells used in the pharmaceutical industry should be appropriately identified and confirmed as original Caco-2 cells. Pharmaceutical regulatory authorities may require documentation of the origin of cells and their genetic properties [10,11]. However, the documents proving the authenticity of the Caco-2 cell line are not strictly specified and are subject to the requirements of the relevant regulatory authority. However, it is common practice to provide documents not older than three years for all continuous human cell lines. For the Caco-2 cell line obtained from a commercial source such as the ATCC or ECACC, relevant purchase orders or invoices may be presented for authentication. Moreover, the authenticity of the internal Caco-2 cell line can be confirmed using STR profiling (ang. short tandem repeats) [52].

**Table 3:** List of commonly used Caco-2 cell lines in permeability studies.

Organization Name	Caco-2 Cell Line Catalog Number	Growth Medium [Original Protocol]	Incubation Conditions	Subject of Scientific Research
American Type Culture Collection (ATCC)	HTB-37™	Eagle’s Minimum Essential Medium with fetal bovine serum (20% final concentration)	95% Air, 5% CO_2_; 37 °C	Classification of drug into BCS group [19,26,35,38,53,54,55] Drug absorption prediction [56,57] nutrient absorption studies [58,59] Toxicity screening [60,61]
European Collection of Authenticated Cell Cultures (ECACC)	86010202	EMEM (EBSS) with 2 mM glutamine, 1% non-essential amino acids (NEAAs), and 10% fetal bovine serum (FBS)	95% Air, 5% CO_2_; 37 °C	Classification of drug into BCS group [42] Drug absorption prediction [62,63] Toxicity screening [64]
Deutsche Sammlung von Mikroorganismen und Zellkulturen (DSMZ)	ACC 169	80% MEM (with Earle’s salts) with 20% heat-inactivated FBS and 1× NEAA	95% Air, 5% CO_2_; 37 °C	Permeability study [65] Nutrient absorption studies [66] Toxicity screening [67]

Each of the presented Caco-2 cell lines is widely used in research related to the absorption and transport of substances through the epithelium of the small intestine. Therefore, the selection of the Caco-2 cell line for permeability studies depends on the researcher’s preference. However, the Caco-2 cell lines delivered from the ATTC are most frequently used in formal studies to classify substances into BCS groups.

A review of the literature revealed that there are no available comparisons of the Caco-2 cell lines obtained from different cell banks in the context of the validation of this in vitro model for pharmaceutical industrial purposes. However, Yasuda et al. showed slightly different α-defensin 5 secretion levels in differentiated Caco-2 cells obtained from the ATCC and the DSMZ. However, both Caco-2 cell lines were considered models for screening healthy food components and drugs that affect α-defensin 5 secretion [68].

### 4.2. General Conditions for the Cultivation of the Caco-2 Cell Line

A literature review showed that the composition of the culture medium for the Caco-2 cell line varies depending on the supplier, the internal culture protocol, and the purpose of the experiment. However, in general, the composition of the culture medium for the Caco-2 cell line contains the basic components necessary for the growth and maintenance of the cells in culture. Typically, Dulbecco’s Modified Eagle’s Medium (DMEM) culture medium supplemented with 10–20% fetal bovine serum (FBS) is used. Some culture media for the Caco-2 line may contain amino acids (1% nonessential amino acid mixture) that are essential for cell growth [19,31,33,35,38]. To prevent infections, antibiotics are added to the media, mainly with amino acids (e.g., 2 mM L-glutamine, 100 U/mL penicillin, and 0.1 mg/mL streptomycin solution) [69,70]. The literature’s data indicate that glutamine is an essential factor in differentiating Caco-2 cells and maintaining the intestinal barrier function. As demonstrated, glutamine maintains the transepithelial resistance of the Caco-2 monolayer and reduces its permeability [71,72,73,74].

The complete culture medium is stored at 4 °C, while the medium additives are stored in small aliquots at −20 °C (to reduce freeze–thaw cycles). The Caco-2 cells are cultured at 37 °C in a humidified atmosphere containing 5% CO_2_ and 95% air [75]. It is common laboratory practice to replace the growth medium every other day. Caco-2 cells are passaged when they reach approximately 80–90% confluence. Passaging the Caco-2 cells in the indicated confluence helps to keep the cells in the exponential growth phase, which is beneficial for maintaining their properties [76,77].

According to the pharmaceutical requirements, internal procedures for the cultivation and maintenance of the Caco-2 cell line should be developed [49]. Additionally, all reagents used to cultivate the Caco-2 cell line should be monitored and reported on an ongoing basis. Data should include the name of the reagent, batch/lot number, source/origin, concentration (if applicable), retest date or expiry date, and storage conditions [78].

### 4.3. Number of Passages of the Caco-2 Cell Line

The influence of factors such as culture conditions and the number of passages on the morphology and biochemistry of the Caco-2 cell line has been reported [16,74,79]. The Caco-2 cell line is characterized by a heterogeneous population demonstrating, e.g., different transport velocity and differentiation parameters, including shaping the brush border. Long-term maintenance of the Caco-2 cell culture, and thus increasing the number of passages, leads to the selection of faster-growing subpopulations of cells expressing different subsets of characteristics [16,79,80,81,82]. The literature review showed that the number of passages has a strong effect on the differentiation process and the integrity of the Caco-2 monolayer (assessed using the TEER value) [35,79,83,84]. Selected permeability studies taking into account the number of passages of the Caco-2 cell line are presented in Table 4.

The presented data show that the Caco-2 cell line is used for permeability studies with a passage number of approximately 20–50 [26,31,38,42]. Moreover, validation of the Caco-2 cell line used for pharmaceutical purposes is performed for a single passage number [26,42]. A review of the literature data showed that active Caco-2 cell cultures should be maintained for a period not longer than three months. The expended culture time and associated cell population drift may affect comparisons with historical permeability data. In addition, every time a new batch of Caco-2 cells is thawed, a new permeability calibration curve must be generated (using model drugs for each group’s permeability [26]).

## 5. Caco-2 Monolayer Suitability

### 5.1. Polarization of Caco-2 Cells on Tissue Culture Inserts

For the transport monolayer preparation, the Caco-2 cells are cultured on membrane inserts made from polycarbonate, polyester, or polyethylene terephthalate. Transparent filters are recommended for monitoring the differentiation process in an inverted or confocal microscope [86]. Depending on the experimental protocol, the Caco-2 cells are seeded in multi-well plates (6, 12, 24, or 96-well) and cultured for 21–25 days [69,87,88,89]. Long-term culture of Caco-2 cells leads to their spontaneous differentiation into mature enterocyte-like cells resembling enterocytes of the small intestine in vivo [69,90,91]. Differentiated Caco-2 cells exhibit functional tight junctions between the neighboring cells and well-developed microvilli on the apical surface [73]. The Caco-2 cell monolayer’s integrity is assessed with transepithelial electrical resistance (TEER) measurements, performed in a non-invasive manner using ohmic resistance [92]. The TERR values in the Caco-2 monolayers from several permeability tests are shown in Table 5.

The TEER vales in the range of 500–1100 ohms are considered acceptable for fully differentiated cultures [92]. However, the data presented (Table 5) show that Caco-2 monolayers with a TERR of at least 300 ohms are commonly used for permeability studies.

According to the biowaiver guidelines, the integrity of the Caco-2 monolayer should be confirmed every time before and after permeability tests [10,11]. The permeability values of the tested compounds should be reported only if the TEER after the transport experiment is at least 75% of the initial value (before the experiment) [86]. Additionally, selecting monolayers with similar TEER values (measured before the permeability test started) is useful in preventing significant variations in the determination of transport rates due to unintended overgrowth [96]. Moreover, the literature’s data indicate the possibility of increasing the performance of the Caco-2 in vitro model by using the same monolayer for further permeation tests. To restore the integrity of the Caco-2 monolayer after permeability tests, two days of incubation in a culture medium are necessary. The presented protocol allows for two additional permeability determinations to be performed using the same Caco-2 monolayer [63].

### 5.2. Permeability Tests across the Caco-2 Monolayer

The permeability of compounds across the Caco-2 monolayer is carried out in two transport directions: apical-to-basolateral (A–B) and basolateral-to-apical (B–A). The compounds are dissolved in the transport buffer and applied to the appropriate compartment in accordance with the tested transport direction [26,31,35]. Permeability studies are commonly performed in HBSS [19] and HEPES [26], and MES [97] or PBS [98] are used less frequently. The transport experimental conditions should mimic the actual physiological environment that drug molecules may encounter in the gastrointestinal tract. Therefore, transport buffers with a pH of 6.5 (duodenum and jejunum) and 7.4. (ileum and colon) are commonly used for permeability tests [19,31,42]. However, Lee and al. showed that a transport buffer with a pH of 7.4 is a good qualitative predictor for physiological intestinal permeability from the duodenum to the colon [99].

Transport experimental conditions should be established on the basis of individual preliminary experiments for each tested drug. However, the transport time should end before the drug concentration in the receiver compartment has reached the equilibrium concentration of the system or before the total amount of drug has been removed by the sampling. The time of transport testing should be adjusted to the physicochemical properties of substances, including their lipophilicity and the number of hydrogen bond atoms. Commonly, the sampling time ranges from 5 min (more lipophilic substance) to 1–2 h (more hydrophilic substance). Sampling intervals should be selected such that the transport experiment ends before the concentration in the receiver exceeds 10% of the donor concentration per time interval [17].

Permeability tests are generally performed in at least three repetitions [10,11,19,20,31,43]. Based on the standardization of the Caco-2 cell line, a positive control (a drug from the high-permeability group) and a negative control (drugs with low and moderate permeability) should be selected. Then, in order to demonstrate the suitability of the Caco-2 model, permeability studies of the tested compound should be performed in the presence of selected control drugs. For this purpose, it is recommended to use multi-well plates or to perform subsequent tests on the same Caco-2 monolayer. The positive and negative controls selected for permeability testing should not exhibit any significant physical, chemical, or permeation interactions with the tested drug [10,11].

Moreover, for regulatory validation purposes (e.g., BCS classification), an additional high-permeability internal standard (HP-IS) and low-permeability internal standard (LP-IS) should be established. The HP-IS is the substance from the high-permeability group which in the validation tests has the lowest P_app_ value (from among at least five tested compounds in this permeability group). Similarly, the LP-IS is the substance from the low-permeability group that has the highest P_app_ value in the validation tests. These additional standards define the lower limit of high permeability (HP-IS) and the upper limit of low permeability (LP-IS), from which the permeability of the tested compound is assessed. Thus, the test drug is classified as a highly permeable substance when its P_app_ value is equal to or greater than P_app_ of the selected HP-IS [100].

The selected transport conditions across the Caco-2 monolayer from scientific and regulatory studies are shown in Table 6.

The in vitro transport studies based on the protocols described by Tavelin [17] and Hubatsch [18] are widely used in permeability research. However, the transport protocols may require modification due to the properties of the tested compounds and the heterogeneity of the Caco-2 cell line [17,18]. As previously mentioned, the use of the Caco-2 cell line in the pharmaceutical industry requires the development of an experimental protocol that will ensure the efficiency, consistency, and reliability of the analytical methods used. Therefore, the conditions for permeability tests should be determined based on preliminary transport experiments. However, the presented data (Table 6) indicate that the transport time across the Caco-2 monolayer does not exceed three hours. Therefore, for preliminary tests, the transport time can be set at two hours, with samples taken at intervals of 15 to 30 min. Furthermore, to establish transport conditions through the Caco-2 monolayer, preliminary experiments can be performed only in one direction (B–A) in a transport buffer at a pH of 7.4 [99].

## 6. Summary

Due to the significant increase in permeability studies using the Caco-2 cell line in recent years, the need to standardize this biological model seems justified. In summary, in this review, we presented the main aspects to consider for the standardization and validation of the Caco-2 cell line in relation to the pharmaceutical guidelines. The combination of the culture conditions, he number of passages, cell differentiation, and monolayer transport conditions has a decisive influence on the development of the monolayer for further suitable permeability studies. Therefore, the development of intra-laboratory protocols for the cultivation and differentiation of Caco-2 cells is necessary to provide consistently cultivated functional cell monolayers and reduce in-house variability. The main conclusions concerning Caco-2 cell line cultivation and permeability test conditions are described in Table 7.

The aspects described in Table 7 may help develop protocols for preparing the cell line for validation purposes (regulatory studies), as well as for permeability studies across biological membranes (scientific studies). Although the Caco-2 model, like any biological model, has several limitations, mainly concerning cell line heterogeneity and external variability, its appropriate standardization may provide the most controlled conditions for establishing compound permeability.

## Figures and Tables

**Table 1:** Model drugs based on biowaiver guidelines with selected experimental results of Caco-2 monolayer permeability and human absorption.

Permeability Group	BCS Model Drug	P_app_ ^1^ [×10^−6^ cm/s]	f_a_ ^2^ [%]	Reference
High-Permeability (f_a_ ≥ 85%)	Antipyrine	76.71 ± 3.59	100	[19] ^3^, [20] ^4^
	Caffeine	44.29 ± 5.12	99	[19] ^3^, [21] ^4^
	Ketoprofen	26.47 ± 4.61	95	[19] ^3^, [22] ^4^
	Naproxen	60.06 ± 2.12	99	[19] ^3^, [23] ^4^
	Theophylline	50.90 ± 3.61	100	[19] ^3^, [20] ^4^
	Metoprolol	37.33 ± 3.82	102	[19] ^3^, [24] ^4^
	Propranolol	30.76 ± 1.91	100	[19] ^3^, [25] ^4^
	Carbamazepine	41.75	98	[26] ^3^, [27] ^4^
	Phenytoin	32.7	90	[26] ^3^, [28] ^4^
	Disopyramide	14.4 ± 2.6	90	[29] ^3^, [30] ^4^
	Minoxidil	13.0 ± 2	95	[31] ^3^, [32] ^4^
Moderate-Permeability (f_a_ = 50–84%)	Chlorpheniramine	16.0	50	[33] ^3^, [34] ^4^
	Creatinine	7.70 ± 0.34	80	[35] ^3^, [36] ^4^
	Terbutaline	2.38	60	[26] ^3^, [37] ^4^
	Hydrochlorothiazide	1.81	70	[26] ^3^, [20] ^4^
	Enalapril	3.5 ± 0.5	60	[38] ^3,4^
	Furosemide	1.29	60	[26] ^3^, [20] ^4^
	Metformin	7.74	60	[26] ^3^, [39] ^4^
	Amiloride	4.29	50	[26] ^3^, [40] ^4^
	Atenolol	1.64	50	[26] ^3^, [20] ^4^
	Ranitidine	2.51	50	[26] ^3^, [41] ^4^
Low-Permeability (f_a_ < 50%)	Famotidine	0.61 ± 0.11	45	[42] ^3,4^
	Nadolol	0.62 ± 0.18	35	[19] ^3^, [20] ^4^
	Sulpride	0.39 ± 0.054	36	[43] ^3^, [24] ^4^
	Lisinopril	0.66	29	[26] ^3^, [44] ^4^
	Acyclovir	0.74 ± 0.13	23	[42] ^3,4^
	Foscarnet	0.35 ± 0.11	17	[35] ^3^, [24] ^4^
	Mannitol	0.19 ± 0.014	26	[43] ^3^, [24] ^4^
	Chlorothiazide	0.71 ± 0.05	20	[42] ^3^, [45] ^4^
	Polyethylene glycol 400	no data	0	-
	Enalaprilat	0.27 ± 0.05	10	[42] ^3^, [46] ^4^
Zero-Permeability	FITC-Dextran	n/a		
	Polyethylene glycol 400	n/a		
	Lucifer yellow	n/a		
	Inulin	n/a		
	Lactulose	n/a		
Efflux Substrates	Digoxin	n/a		
	Paclitaxel	n/a		
	Quinidine	n/a		
	Vinblastine	n/a		

^1^ P_app_—data from selected Caco-2 permeability experiments; values represent the mean and standard deviation (if available). ^2^ f_a_—% fraction dose absorbed in humans. ^3^ reference for P_app_ values. ^4^ reference for human absorption (f_a_). n/a = not applicable.

**Table 2:** The effect of selected BCS model drugs on Caco-2 cell viability [MTT test].

BCS Model Drug	Time Exposed [h]	Concentration [µM]	Caco-2 Cell Viability [%]	Reference
Antipyrine	24	25–500	≥100	[49]
Ketoprofen	24	25–300	≥100	
Digoxin	24	5	≥100	
		50 or high	≤75	
Famotidine	Not available	80–1250	≥90%	[50]
		5000 or high	≤60	

**Table 4:** Number of passages of the Caco-2 cell line in various permeability studies.

Purpose of the Study	Number of Passage	Reference
Validation of 96-well Caco-2 screening assay for predicting in vivo intestinal absorption in the drug discovery stage	19	[26]
Intestinal permeability study of Minoxidil	39–40	[31]
In vitro evaluation of absorption characteristics of Peramivir for oral delivery	33–35	[85]
Variety of experimental systems to evaluate the suitability of enalapril as a model compound	25–35	[38]
Demonstrating experimental suitability of the Caco-2 cell model for BCS-based biowaiver	47	[42]

**Table 5:** The TEER values in the Caco-2 monolayers after 21 days of culture under various experimental conditions.

Purpose of the Study	Cell Culture Plates	Seeding Density [cm/s]	Time Differentiation [days]	TEER [Ohm × cm^2^]	Reference
Evaluation of the cytotoxic effect and permeability of ziprasidone hydrochloride monohydrate	6-well plate	7 × 10^−5^	21	>300	[93]
Permeability tests in a 12-well plate as a reference for a new rapid 96-well protocol	12-well plate	0.75 × 10^−5^	21	600 ± 70	[94]
Assessment of permeability of Chamaelirium luteum (false unicorn) open-chain steroidal saponins	24-well plate	7 × 10^−5^	21	>300	[95]
Validation of Transwell-96 plates for high-permeability screening	96-well plate	8 × 10^−5^	21	>400	[89]

**Table 6:** Selected transport conditions across Caco-2 monolayer in the apical-to-basolateral direction from scientific (permeability studies, S) and regulatory (BCS classification, R) studies.

Purpose of the Study	Culture Plate	Transport Buffer	Volume of Apical Compartment [mL]	Volume of Receiver Compartment [mL]	Time Transport [h]	Time Point [min]	Sample Volume [mL]	Reference
Demonstrating suitability of the Caco-2 cell model for BCS-based biowaiver (R)	no data	HBSS pH = 7.4	0.4	0.8	3	30, 60, 90, 180	0.07	[42]
Classification of metaxalone into BCS group (R)	12-well plate	HEPES pH = 7.4	0.5	1.5	2	20, 40, 60 or 60, 40, 80	0.2	[101]
Permeability across Caco-2 of apomorphine (S)	48-well plate	HBSS pH = 6.5 (aplical) HBSS pH = 7.5 (receiver)	0.5	1.0	3	5, 10, 15, 30, 45, 60, 90, 120, 180	0.1	[102]
Intestinal permeability study of Minoxidil (S)	12-well plate	HBSS, HEPES, MES	0.5	1.5	2	15, 30, 45, 60, 90, 120	0.1	[31]

**Table 7:** The main points for the standardization and validation of the Caco-2 cell line in relation to pharmaceutical guidelines and experimental permeability data.

Experimental Stage	Pharmaceutical Requirements	Main Points
Selection of the origin of the Caco-2 cell line	Proof of cell origin required	The Caco-2 cell line delivered from the ATTC—the most commonly used commercial source
2. Selection of compound concentrations for permeability testing	The test substance concentrations should be determined (highest non-cytotoxic concentration)	Based on a balance between solubility, cytotoxicity, and analytical response MTT—the most commonly used cytotoxicity assay
3. Cultivation of Caco-2 cell line	Internal procedures for cultivation and maintenance of the Caco-2 cell line	Basic culture medium
(i) Number of passages of Caco-2 cell line	The Caco-2 culture conditions should be determined (number of passages)	Approx. 20–50 passages Validation performed with the lowest possible passage number difference The active caco-2 cell cultures maintained not longer than three months
(ii) Polarization of Caco-2 cells	Integrity of Caco-2 monolayer should be confirmed using TEER measures	Caco-2 monolayer with a TEER value above 300 ohm × cm^2^ TEER value after the transport experiment—at least 75% of the initial value (before the investigation)
4. Permeability tests across Caco-2 monolayer	Correlation between P_app_ and f_a_ for selected model drugs	Five model drugs from each permeability group (formal regulatory studies) Established positive and negative internal standards (regulatory and scientific studies) Established HP-IS and LP-HS for regulatory purpose (formal regulatory studies)

## Data Availability

Not applicable.

## Abbreviations

ATCC	American Type Culture Collection
A-B	Apical-t-o-basolateral
B-A	Basolateral-to-apical
BA	Bioavailability
BCS	Biopharmaceutics Classification System
DMEM	Dulbecco’s Modified Eagle Medium
DSMZ	Deutsche Sammlung von Mikroorganismen und Zellkulturen
EBSS	Earle’s Balanced Salt Solution
ECACC	European Collection of Authenticated Cell Cultures
EMA	European Medicines Agency
EMEM	Eagle’s minimum essential medium
f_a_	% fraction dose absorbed in humans [%]
FBS	Fetal bovine serum
FDA	Food and Drug Administration
HEPES	4-(2-hydroxyethyl)-1-piperazineethanesulfonic acid
HP-IS	High-permeability internal standard
LP-IS	Low-permeability internal standard
MES	2-(N-morpholino)ethanesulfonic acid
MTT test	3-(4,5-dimethyl-2-thiazolyl)-2,5-diphenyl-2H-tetrazolium bromide test
NEAA	Non-essential amino acids
P_app_	The apparent permeability coefficients [cm/s]
PBS	Phosphate Buffered Saline
TEER	Transepithelial electrical resistance
